# An Evolvable Organic Electrochemical Transistor for Neuromorphic Applications

**DOI:** 10.1002/advs.201801339

**Published:** 2019-02-04

**Authors:** Jennifer Y. Gerasimov, Roger Gabrielsson, Robert Forchheimer, Eleni Stavrinidou, Daniel T. Simon, Magnus Berggren, Simone Fabiano

**Affiliations:** ^1^ Laboratory of Organic Electronics Department of Science and Technology Linköping University SE‐601 74 Norrköping Sweden; ^2^ Department of Electrical Engineering Linköping University SE‐581 83 Linköping Sweden

**Keywords:** conducting polymers, evolvable electronics, neuromorphic, organic electrochemical transistors, organic electronics

## Abstract

An evolvable organic electrochemical transistor (OECT), operating in the hybrid accumulation–depletion mode is reported, which exhibits short‐term and long‐term memory functionalities. The transistor channel, formed by an electropolymerized conducting polymer, can be formed, modulated, and obliterated in situ and under operation. Enduring changes in channel conductance, analogous to long‐term potentiation and depression, are attained by electropolymerization and electrochemical overoxidation of the channel material, respectively. Transient changes in channel conductance, analogous to short‐term potentiation and depression, are accomplished by inducing nonequilibrium doping states within the transistor channel. By manipulating the input signal, the strength of the transistor response to a given stimulus can be modulated within a range that spans several orders of magnitude, producing behavior that is directly comparable to short‐ and long‐term neuroplasticity. The evolvable transistor is further incorporated into a simple circuit that mimics classical conditioning. It is forecasted that OECTs that can be physically and electronically modulated under operation will bring about a new paradigm of machine learning based on evolvable organic electronics.

## 1 Introduction

Researchers have drawn analogies between computers and the mind since the age of the Turing machine.^1^ Unlike the binary logic of conventional silicon‐based circuitry, however, the brain relies on the adjustment of synaptic weights to integrate complex, parallel, and multidimensional stimuli to perform computations, which then instruct appropriate response actions.^2^

The strategy of assigning a system of weights to inputs has already been implemented by artificial neural networks operating at the software level.^3^ Digital hardware systems that resemble the computational features of neural networks, however, require hundreds of transistors to simulate just a single neuron and are therefore difficult to scale up due to power constraints. A more direct analogy to neural processing has been drawn between analog memristor‐type synaptic mimics.^4^ Like the synapse, the memristor is a two‐terminal device that can access a range of weights and collapse the functions of memory and processing to a single element.^5^ A far less explored synaptic mimic was first published in 1960 in the form of a memistor—a three‐terminal device wherein the conductivity across a resistor is modulated by reversible electroplating of a more conductive metal on the resistor surface.^6^ While the memistor is a very interesting early exploration of machine learning, each device requires the encasement of a highly concentrated sulfuric acid solution in glass and thus presents great difficulties for miniaturization, mass production, and compatibility with the established microfluidic platform as well as with emerging liquid microfabrication technologies.^7^ Analog electronic devices simulate neuroplasticity by exhibiting either an increase, referred to as potentiation, or a decrease, referred to as depression, in the sensitivity of the output signal to the stimuli delivered at the input terminal.[[qv: 4a,d,e]] A device that encompasses both of these behaviors at the short‐term and long‐term timescales is important for developing the next generation of neuromorphic devices.

Organic electrochemical transistors (OECTs) are the most recent addition to the neuromorphic device toolbox, having recently been shown to exhibit memory functionalities like long‐term plasticity, spike‐timing dependent plasticity, and homeostatic plasticity.^8^ The existing technology, however, still relies on prefabricated circuitry that can be adjusted through supervised or unsupervised learning to accomplish a given task. To date, the brain remains unique in the sense that it possesses the capability to make new connections where none existed before.

**Figure 1:**
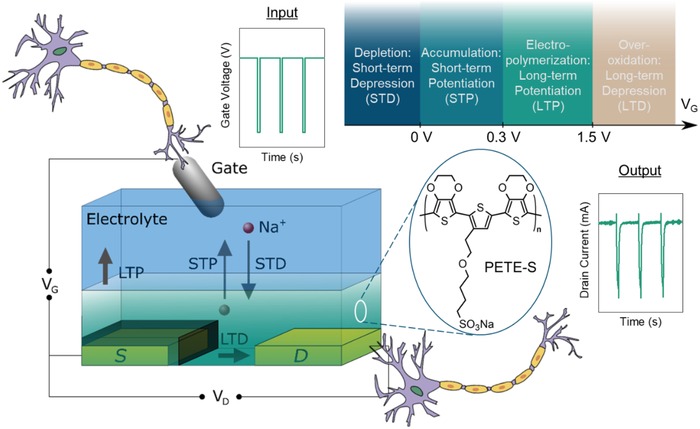
Synaptic transistor. Sketch of the organic electrochemical transistor, formed by electropolymerization of ETE‐S in the transistor channel. The electrolyte solution is confined by a PDMS well (not shown). In this work, we define the input at the gate as the presynaptic signal and the response at the drain as the postsynaptic terminal. During operation, the drain voltage is kept constant while the gate is pulsed. Synaptic weight is defined as the amplitude of the current response to a standard gate voltage characterization pulse of −0.1 V. Different memory functionalities are accessible by applying gate voltage spikes at different ranges or timing intervals.

Here, we report an evolvable OECT that is formed in situ by electropolymerizing a self‐doped conjugated monomer, sodium 4‐(2‐(2,5‐bis(2,3‐dihydrothieno[3,4‐b][1,4]dioxin‐5‐yl)thiophen‐3‐yl)ethoxy)butane‐1‐sulfonate (ETE‐S), as the transistor channel (**Figure** **1**). The OECT that incorporates PETE‐S as the channel material operates in the hybrid accumulation–depletion mode. Thus, the drain current (*I*_D_) can be modulated to different extents, either positively or negatively, depending on the magnitude and polarity of the gate voltage (*V*_G_). As others have done, we find it useful to draw an analogy between the electrochemical transistor and the synapse in which the gate behaves as the presynaptic terminal, the channel determines the synaptic weight, and the drain serves as the postsynaptic terminal. Thus, an input voltage spike from the gate is weighted by the channel conductance and is subsequently converted into an output current spike at the drain that is proportional to the synaptic strength. Fabrication by means of electropolymerization allows for a stimulus‐driven formation of new electronic synapses, which, in the biological sphere, is a major contributor to neuroplasticity. Lasting changes in channel properties can be achieved by either growing additional channel material to enhance conductance or overoxidizing the channel to reduce conductance, which is analogous to long‐term potentiation (LTP) and long‐term depression (LTD), respectively, of synaptic weight. Using this approach, the conductance of a channel can be modulated by orders of magnitude.

Transistors based on the polymerized ETE‐S material are additionally amenable to transient modification of channel properties through the ionic doping and dedoping of the channel, i.e., ion exchange drives the accumulation and depletion of hole charge carriers in the channel. Paired pulse facilitation is evident when presynaptic spikes arrive at time intervals shorter than the spike current decay time, leading to an accumulation of anions in the channel. In contrast, short‐term depression (STD) is observed when the channel is depleted of anions by applying a positive gate potential prior to a presynaptic spike.

We further incorporate the synaptic transistor into an evolvable learning circuit, taking advantage of its unique capacity to form and strengthen new connections. We are thus able to model classical (Pavlovian) conditioning, a widely studied biological learning process that induces the linkage of a response to a neutral stimulus through simultaneous presentation of the neutral stimulus with a stimulus that already elicits the desired response. This mode of learning has previously been modeled using a prefabricated OECT by van de Burgt et al. using a conventional relay to change the doping state of the channel.[[qv: 8a]] Due to the chemically and electrically stable nature of the electropolymerized PETE‐S, we are able to bypass the relay entirely, reducing the circuit only to the synaptic elements.

## 2 Results

### 2.1 Initiation of Channel Growth

**Figure 2:**
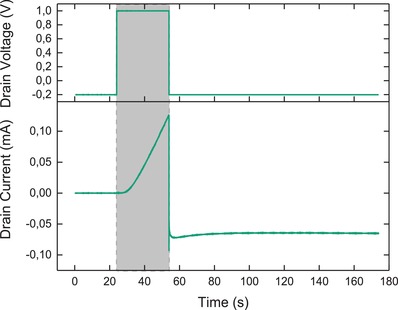
Channel initiation. Time‐dependent plot of the *V*_D_ (top plot) and *I*_D_ (bottom plot) before, during, and after initiation of the transistor channel. The channel is initiated by applying a *V*_D_ of 1 V for 30 s (marked in gray) and characterized before and after initiation at a *V*_D_ of −0.2 V.

In order to fabricate a transistor channel on a set of prepatterned gold electrodes, we electropolymerize the ETE‐S monomer at the drain, allowing it to grow until it makes an electrical connection with the source. By applying a positive drain voltage (*V*_D_) of 1 V for 30 s, we obtain a robust connection between the source and drain. During the initiation, a *V*_D_ of −0.2 V is used to evaluate conductivity between the source and the drain before and after the growth of the channel. The channel forms within seconds of applying an oxidizing *V*_D_, as evidenced by the sharp increase in the *I*_D_ (**Figure** **2**). This example is given to demonstrate the linear nature of channel growth. To demonstrate learning behaviors, however, low conductance in the initial state is a more suitable mimic of the unconditioned synapse. It is thus useful that formation of the contact between the source and the drain can be observed in real‐time, whereupon the *V*_D_ can be switched off.

### 2.2 OECT Characteristics

**Figure 3:**
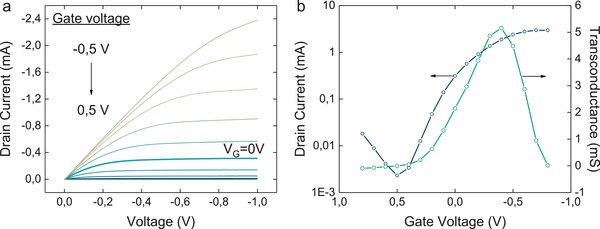
OECT characteristics. a) Output characteristics of the PETE‐S OECT at gate voltages ranging from −0.5 to 0.5 V in 0.1 V intervals suggest that PETE‐S is partially self‐doped and can thus be operated in both the accumulation and depletion modes. b) Transfer characteristics and transconductance of the PETE‐S OECT at a *V*_D_ of −1 V. All characterizations were performed in an aqueous solution of 10 × 10^−3^ m NaCl.

The PETE‐S OECT channel was fabricated on a silicon oxide substrate patterned with source and drain electrodes (30 µm channel length; 1 mm channel width). The morphology, spectroelectrochemistry, and nature of charge carriers for the water‐soluble ETE‐S have been evaluated in prior publications.^9^ By applying an oxidizing potential at the drain in a solution of ETE‐S monomer, the ETE‐S is electropolymerized at the electrode surface, protruding until PETE‐S makes contact with the source electrode. Devices are characterized in an aqueous solution of 10 × 10^−3^ m NaCl, which is confined in a PDMS well over the source and drain electrodes. An Ag/AgCl pellet is immersed in the electrolyte and used as the gate. The output curve shows that the PETE‐S OECT can be operated in a hybrid accumulation/depletion mode because the intrinsic sulfonate group is insufficient to fully dope the channel (**Figure** **3**a). Since the OECT channel is hydrated, it permits the penetration of ions from the electrolyte into the bulk of the channel volume. The application of a negative *V*_G_ results in the simultaneous migration of anions into the channel and the extraction of cations that compensate the charge on the sulfonate from the channel, which leads to the ionic doping of the channel material and a consequent increase in *I*_D_. A cyclic voltammogram of a PETE‐S layer was obtained by using the drain, a Pt wire and a Ag/AgCl pellet as the working, counter, and reference electrode, respectively (Figure S1a, Supporting Information). The large capacitive currents observed at electrode potentials greater than −0.8 V confirms that PETE‐S is a p‐type semiconductor. The transfercurve, obtained at a *V*_D_ = −1 V, highlights the hybrid accumulation–depletion mode of operation of our OECT (Figure 3b). The *I*_D_ is suppressed by 99% when *V*_G_ = 0.5 V and is enhanced by 850% as *V*_G_ = −0.5 V is applied, when comparing to the *I*_D_ at *V*_G_ = 0 V. The transconductance, which peaks at 5.2 mS at a *V*_G_ of −0.4 V, indicates that the PETE‐S is an excellent channel material besides its neuromorphic properties. As earlier reported for OECTs^10^ the device characteristics, such as the *I*_D_ level, are dependent on the dimensions of the transistor channel. This includes the thickness, which here is variable in situ under specific electric addressing conditions. The transistor characteristics described are thus representative of this class of transistors and do not apply to each device presented in this study.

### 2.3 Long‐Term Plasticity

Unlike most neuromorphic systems described to date, the nervous system operates in the time/frequency domain where neuronal activation triggers potential spikes of equal magnitude at a variable rate.^11^ The strength of a synapse, then, is proportional to the probability of the presynaptic cell triggering a signal at the postsynaptic cell. When implemented on the electronic platform, however, it is much more convenient to treat synaptic strength as a numerical weight.[[qv: 4a,e,h,5,8a,12]] Here, we define the synaptic weight as the amplitude of the *I*_D_ response to a standard *V*_G_ characterization spike of −0.1 V. Such characterization spikes are used to evaluate the response of the synaptic transistor to stimuli that induce both potentiation and depression. Long‐term plasticity is a term used to describe enduring changes in synaptic strength, or the sensitivity of the postsynaptic neuron to input from the presynaptic terminal.^13^ Long‐term plasticity can lead to an increase or a decrease in synaptic strength in the form of potentiation (LTP) or depression (LTD), respectively. In the brain, LTP and LTD are induced by specific patterns of synaptic activity,^13^ which we can simulate on the PETE‐S OECT by sending conditioning spikes to the gate that lead to enduring changes in the amplitude of the characterization spikes generated by the device.

**Figure 4:**
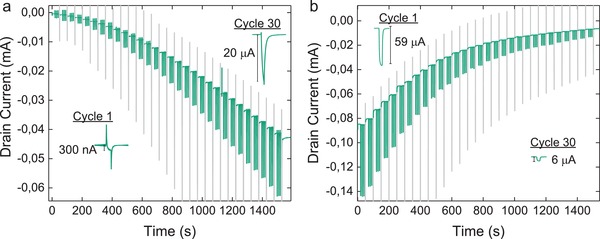
Long‐term plasticity. a) Long‐term potentiation is induced by applying 30 *V*_G_ spikes of −0.5 V (gray) while b) long‐term depression is induced by applying *V*_G_ spikes of −2 V (gray). Spikes that do not result in electropolymerization (*V*_G_ = −0.1 V, green) are used to characterize the strength of the synaptic transistor.

In order to demonstrate LTP of the transistor signal, we first induce the formation of a low‐conductance channel by applying a *V*_D_ pulse that is sufficient to create a weak contact between the source and the drain. Further electropolymerization at the channel can also be induced by applying a sufficiently negative *V*_G_. We investigate effects of *V*_G_ on *I*_D_ by applying pulses at an initiated channel (Figure S2, Supporting Information). A significant increase in baseline *I*_D_ is observed after each set of pulses when *V*_G_ ≤ −0.3 V. The Δ*I*_D_ peaks at −0.6 V and exhibits a downward trend at more negative voltages as a result of the concurrent overoxidation of the channel material. We thus use conditioning *V*_G_ spikes of −0.5 V to trigger stepwise channel growth at an initiated channel (**Figure** **4**a). Between conditioning spikes, characterization spikes (*V*_G_ of −0.1 V for 1 s) are used to monitor the strengthening of the connection between the source and drain. In addition to an increasing baseline current, the amplitude of the characterization spikes increases from an initial 300 nA to 20 µA after the 30th conditioning pulse, thus resulting in a net increase of the enduring synaptic strength by two orders of magnitude. It should be noted that the transient spikes of opposite sign are observed at channels with low synaptic weight due to the high capacitance of the exposed source and drain electrodes. This effect is diminished as the synaptic weight increases and the source and drain are covered by PETE‐S and can be minimized in future iterations of the device by using an insulating layer that covers the unnecessarily exposed metal.^14^

The reverse process, LTD, is attained by applying negative conditioning voltage spikes to the gate (*V*_G_ of −2 V, 100 ms) in the absence of monomer, which results in overoxidation of the channel (Figure 4b). Overoxidation is observed at *V*_G_ ≤ −1 V (Figure S2, Supporting Information). As with LTP, we can achieve controlled LTD by applying conditioning spikes to the gate. We have found that it is possible to regrow the channel after overoxidation (Figure S3, Supporting Information). Overoxidized material adheres weakly to the substrate and can be washed away by rinsing with water, as shown in the optical micrographs (Figure S4, Supporting Information).

### 2.4 Short‐Term Plasticity

**Figure 5:**
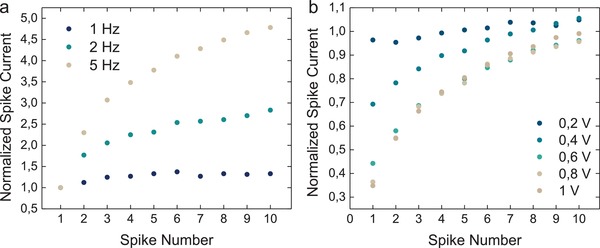
Short‐term plasticity. a) To evaluate short‐term potentiation, a sequence of 10 spikes (100 ms each spike, *V*_G_ = −0.1 V) was applied at a range of frequencies. As spike frequency increases, the amplitude of subsequent spikes grows as ions accumulate within the transistor channel. This accumulation results in higher channel conductance. The spike current is normalized by the current of the first spike in the sequence. b) Short‐term depression is attained by applying a positive voltage of varying magnitude to the gate for 10 s prior to producing a sequence of 10 spikes. The spike current is normalized by the current of the last spike before the positive voltage was applied.

A transient potentiation or depression of characterization spike amplitude can be attained by modulating the ionic concentrations within the OECT channel. We induce a form of short‐term potentiation (STP) by choosing a characterization spike duration that is below what is necessary for anions to completely dope the channel. In this configuration, subsequent characterization spikes lead to the accumulation of anions and result in a higher spike current intensity when the time between spikes is insufficient to allow for anions to return to the equilibrium concentration. This effect can be observed in **Figure** **5**a. Sequences of 10 spikes (100 ms each spike, *V*_G_ = −0.1 V) were applied at different frequencies. At a frequency of 1 Hz, the spike intensity remains constant throughout the spike sequence, indicating that subsequent spikes do not result in the accumulation of anions within the channel. As spike frequency increases, the intensity of subsequent spikes grows as ions accumulate in the channel. This effect results in higher channel conductance. The incomplete diffusion of anions out of the channel is also evidenced in the incomplete decay of the *I*_D_ after each spike at frequencies above 1 Hz (Figure S11, Supporting Information). We estimate an optimized operation energy of ≈1 pJ (Figure S5, Supporting Information), which is similar to the energy consumption reported for other electrochemical neuromorphic organic devices having comparable channel area[[qv: 8a]] and can be minimized by downsizing the device footprint.

STD is achieved by applying an inhibitory stimulus to the gate, which leads to a reduction in channel conductance. By applying a positive *V*_G_, cations are injected into the channel where they compensate for the negative charge on the intrinsic PETE‐S sulfonate group. The sulfonate is thus prohibited from interacting with the charge‐transporting holes in the polymer and the *I*_D_ is switched off. In parallel to the injection of cations, anions from the electrolyte, which serve as counter ions in addition to the sulfonate groups, are expelled from the channel volume. To evaluate STD, we applied two successive sets of 10 characterization spikes. Prior to the second set of characterization spikes, we dedoped the channel by applying a positive gate voltage for 10 s (Figure S12, Supporting Information). The current generated by each spike in the second set is normalized by the last spike from the first set to assess the recovery from the suppression stimulus. The short‐term inhibitory effect of dedoping the channel scales with voltage (Figure 5b).

### 2.5 Classical Conditioning Circuit

**Figure 6:**
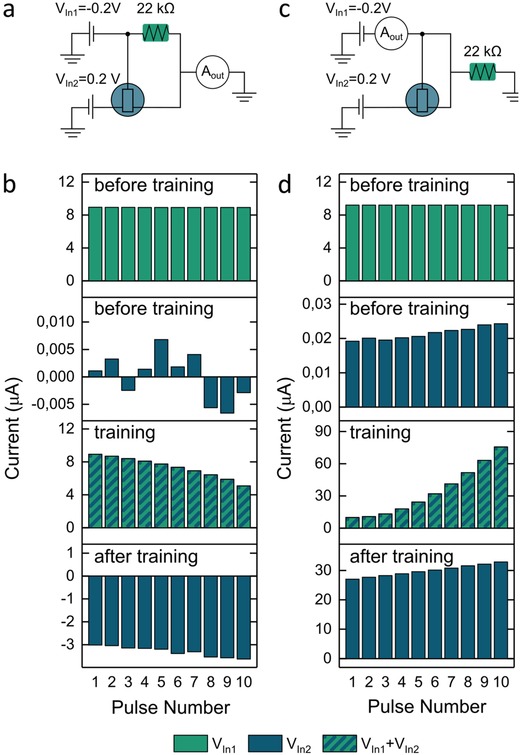
Classical conditioning. a) Diagram of a classical conditioning circuit incorporating a preformed PETE‐S resistor and an evolvable OECT. b) Classical conditioning of the evolvable OECT incorporated in circuit a. Each bar represents the current observed at the end of a *V*_In_ *_X_* pulse of 5 s. Prior to training, *V*_In1_ generates a detectable current pulse at the ammeter whereas the intensity of current generated by the application of *V*_In2_ is well below the noise of the instrument. During training, when *V*_In1_ and *V*_In2_ are applied together, the voltage difference is sufficient to grow the OECT channel. Since *V*_In1_ and *V*_In2_ are of opposing polarity, the growth of the OECT leads to the reduction in the measured output current at the ground terminal. Growth of the OECT channel during training is evidenced by the strong signal generated by *V*_In2_ after training. c) Measuring the current at the terminal where *V*_In1_ is applied, as shown in panel c, causes the current from both inputs to have the same sign. d) Prior to training, just as in panel b, only *V*_In1_ produces a notable current. The low, but detectable current produced by *V*_In2_ is of unknown origin. During training, the current exhibits an upward trend due to the constructive interference from the application of *V*_In1_ and *V*_In2_. After training, the current induced by applying *V*_In2_ again increases by three orders of magnitude. It should be noted that the current observed during training is larger than the simple sum of the currents generated by *V*_In1_ and *V*_In2_ because an effective *V*_D_ of 0.4 V in addition to a *V*_G_ of −0.2 V are applied during training after the OECT channel is formed.

To demonstrate the neuromorphic functionality of the PETE‐S OECT, we construct two simple circuits that model classical conditioning by forming a new connection between the output current and a previously irrelevant input voltage. Both circuits consist of a preformed PETE‐S resistor and an evolvable OECT assembled such that the OECT channel only grows when input voltage 1 (*V*_In1_) and input voltage 2 (*V*_In2_) are simultaneously applied (**Figure** **6**).

When *V*_In1_ is applied to an untrained circuit, the current passes through the resistor to ground and is measured by the ammeter. When *V*_In2_ is applied to an untrained circuit, no current is measured because the source and drain are not yet connected by a channel. Neither *V*_In1_ nor *V*_In2_ provides a sufficient voltage to induce electropolymerization of the channel (Figure S13, Supporting Information). Thus, for both circuits, prior to training, only *V*_In1_ generates a significant current response. During training, the voltage difference between the drain and the gate of the OECT is 0.4 V, which is enough to grow the channel at the drain. Depending on the arrangement of the circuit elements, the inputs produce either destructive (Figure 6b, training) or constructive (Figure 6d, training) interference at the ammeter when operated simultaneously. Through training, the current response to *V*_In2_ increases by three orders of magnitude.

## 3 Discussion

We report an evolvable organic electrochemical transistor that mimics the biological synapse, exhibiting short‐ and long‐term potentiation and depression. We fabricate the transistor channel on a set of prepatterned source and drain electrodes, producing the first synaptic device that can generate new synapses within its working environment similarly to how biological synapses establish, evolve, and operate.

Long‐term changes in the channel conductance are induced by electropolymerization of the monomer to form the transistor channel. Using this fabrication approach, conductance is controlled by the interconnectivity between the source and the drain rather than the geometric volume dictated by the channel dimensions. The range of attainable conductance values can thus span orders of magnitude for a given device at a given set of working conditions (*V*_D_ and *V*_G_). We induce LTD of the channel conductance by sending negative *V*_G_ pulses that lead to the irreversible overoxidation of the PETE‐S. Considering the similarity of the conjugated thiophene backbone of PETE‐S to that of poly(3,4‐ethylenedioxythiophene) (PEDOT), we presume that the mechanisms of overoxidation of these two polymers are also similar and involves the formation of sulfone and carboxyl groups.^15^ After overoxidation, even without the removal of overoxidized material, the channel can be regenerated by elecropolymerizing additional PETE‐S. Since a large portion of the channel is unaffected by overoxidation, subsequent rounds of channel formation require less time to form a connection and result in a channel with higher conductance for a given set of electropolymerization parameters (Figure S3, Supporting Information). The chemical structure of the overoxidized material as well as its morphology and adhesion to the substrate are of great interest to the future development of this system and are currently under investigation.

While long‐term modulation of the channel conductance persists for months (Figure S6, Supporting Information), we further mimic short‐term synaptic plasticity that occurs on a time‐scale of seconds by inducing nonequilibrium doping states in the channel. Facilitated by ion exchange from the electrolyte, STP is observed when the pulse duration is shorter than what is required for saturation of the current and the time interval between pulses is shorter than the decay constant of the current. In this interrogation regime, anions from the electrolyte accumulate in the transistor channel with each successive pulse and compensate for the mobile holes in the PETE‐S material. The reverse of this effect, STD, is attained by sending a positive *V*_G_ pulse which dedopes the channel both by extracting mobile anions from the channel and injecting cations from the electrolyte to pair with the native sulfonate groups on the PETE‐S polymer. Characterization spikes sent after this positive *V*_G_ pulse recover their intensity in proportion to the time constant of the channel to return to its equilibrium doping level. The injection of cations is additionally coupled to the electrochemical reduction of the channel to its nonconducting state just as the withdrawal of anions is coupled to the oxidation of the channel to a highly conducting state. It should be noted that the short‐term memory functionalities that we demonstrate in this work are unique to this material due to its hybrid depletion–accumulation mode of operation. While the hybrid mode has previously been reported for material blends,^16^ precise control over channel composition of a blend would be difficult to achieve via electropolymerization.

As a demonstration of how channel formation and LTP can extend beyond the single synapse, we designed two simple circuits incorporating a preformed PETE‐S resistor and an unformed OECT. In this electronic model of classical (Pavlovian) conditioning, the preformed resistor represents an established synapse whereas the OECT represents an unformed synapse subject to conditioning. The voltage required to induce ETE‐S electropolymerization and form a link between the output and *V*_In2_ is only sufficient during the training stage when both inputs operate simultaneously. The constructive and destructive interference between the inputs may be useful in furthering the sophistication of neuromorphic devices to produce excitatory and inhibitory circuits.

We are confident that this operational scheme can be implemented on a crossbar array for further development. In short, PETE‐S OECTs are singularly suited for neuromorphic applications and constitute a significant advance in the field.

## 4 Experimental Section

*Device Fabrication*: Silicon substrates with a 1 µm thermally grown oxide layer were cleaned by sequential sonication in 2% Hellmanex, DI water, acetone, and isopropanol. Source and drain electrodes (2 nm Cr, 50 nm Au) were thermally evaporated on the substrate using an evaporation mask (Source–Drain Deposition Mask for Low Density OFETs, Osilla Ltd, UK).

A detailed description of ETE‐S synthesis has been reported previously.^9^ A PDMS well, 2 mm in thickness, was hand‐cut to contain the ETE‐S monomer solution (DI water containing 1 mg mL^−1^ ETE‐S and 10 × 10^−3^ m NaCl). Data presented in Figure 3 were collected in the absence of monomer (10 × 10^−3^ m NaCl in DI water).

Several methods can produce functional OECT channels. To obtain results in Figure 3, the *V*_D_ was stepped between 1 and −1 V for 5 s each step until the desired current range was reached. Alternatively, to obtain channels in Figure 4a, at the lower limits of conductivity, a *V*_D_ of 1 V was applied for 5 s, after which the drain voltage was reduced to 0.5 V and applied until the oxidation current showed an upward trend. This configuration produced structures consistent with diffusion‐limited growth (see inset in Figure S7, Supporting Information) while minimizing the overoxidation of the just‐deposited polymer.

*Device Characterization*: The output curve and circuit data were obtained on a Keithley Model 4200 Semiconductor Characterization System (Keithley Instruments, USA). An SP‐300 Bio‐Logic potentiostat/galvanostat in a synchronized two‐channel configuration was used for time‐resolved measurements (Bio‐Logic Science Instruments, France). The source was connected to the ground, counter, and reference leads of both channels while the working leads were connected to the drain and gate electrodes. An Ag/AgCl pellet electrode was used as the gate (Warner Instruments, USA) (Figure S7, Supporting Information). ETE‐S monomer is absent from solution in Figures 3 and 4b.

For spiked LTP, STP, STD, and LTD experiments, the drain voltage was set to −0.2 V while characterization spikes of −0.1 V were applied to the gate. In obtaining STP measurements, the characterization spikes were limited to 100 ms to avoid current saturation. LTP was induced by applying one second *V*_G_ spikes of −0.5 V. LTD was induced by applying 100 millisecond *V*_G_ spikes of −2 V. STD was induced by applying a positive 10 s pulse of varying magnitude, followed by 1 s characterization pulses.

## Conflict of Interest

The authors declare no conflict of interest.

## Supporting information

Supplementary
