# Supplementary material for: An Evolvable Organic Electrochemical Transistor for Neuromorphic Applications
Source: Adv Sci (Weinh). 2019 Feb 4;6(7):1801339. doi: 10.1002/advs.201801339 (PMC6446606; doi:10.1002/advs.201801339)
Supplement: Supplementary file 1 — Supplementary [file ADVS-6-1801339-s001.pdf]

Copyright WILEY-VCH Verlag GmbH & Co. KGaA, 69469 Weinheim, Germany, 2019.

Supporting Information

for *Adv. Sci.,* DOI: 10.1002/advs.201801339

An Evolvable Organic Electrochemical Transistor for Neuromorphic Applications

Jennifer Y. Gerasimov, Roger Gabrielsson, Robert Forchheimer, Eleni Stavrinidou, Daniel T. Simon, Magnus Berggren, and Simone Fabiano**

WILEY-VCH

Copyright WILEY-VCH Verlag GmbH & Co. KGaA, 69469 Weinheim, Germany, 2016.

Supporting Information

An Evolvable Organic Electrochemical Transistor for Neuromorphic Applications

Jennifer Y. Gerasimov, Roger Gabrielsson, Robert Forchheimer, Eleni Stavrinidou, Daniel T. Simon, Magnus Berggren*, Simone Fabiano*

Dr. J. Y. Gerasimov, Dr. R. Gabrielsson, Dr. E. Stavrinidou, Dr. D. T. Simon, Prof. M. Berggren, Dr. S. Fabiano
Laboratory of Organic Electronics, Department of Science and Technology, Linköping University, SE-601 74 Norrköping, Sweden
E-mail: magnus.berggren@liu.se, simone.fabiano@liu.se

Prof. R. Forchheimer
Department of Electrical Engineering, Linköping University, SE-581 83 Linköping, Sweden

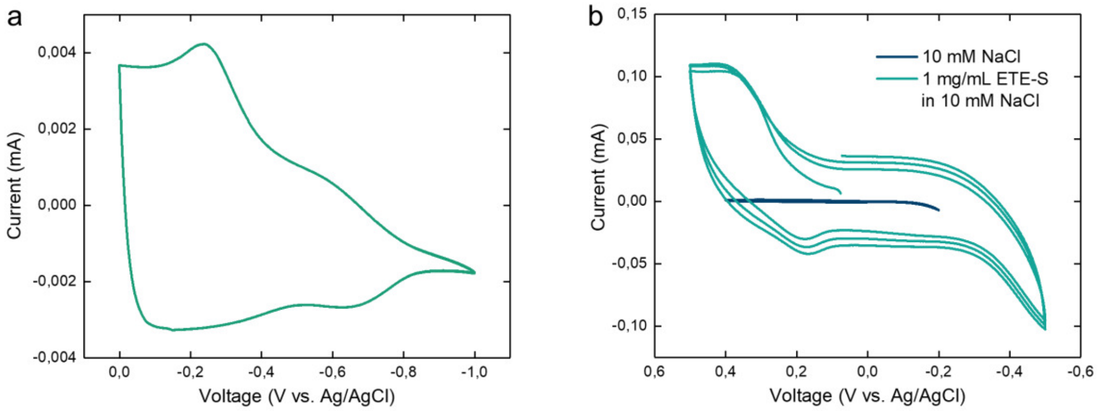

Figure S1. Electrochemical characterization. a) Electrodeposited PETE-S was evaluated with cyclic voltammetry on a gold working electrode using an AgCl wire reference electrode and a platinum counter electrode. b) ETE-S electropolymerization on a gold working electrode by cyclic voltammetry. The background voltammogram (blue) is obtained in 10 mM NaCl background electrolyte and the voltammogram showing electropolymerization (green) is obtained in background electrolyte containing 1 mg/mL ETE-S.

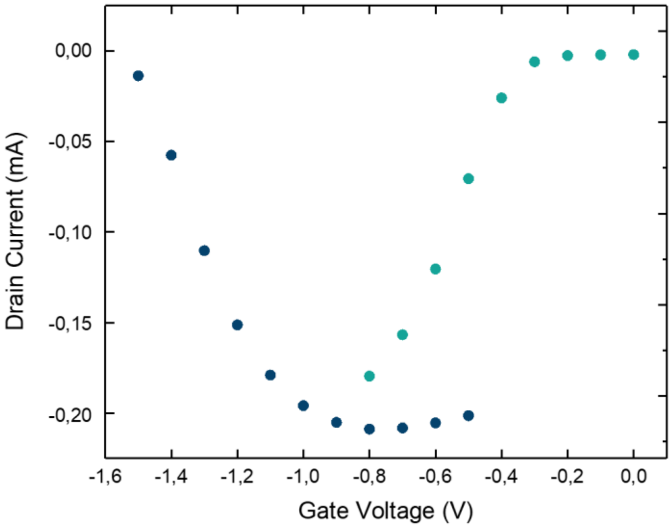

Figure S2. Gate voltage-dependent long-term plasticity. The long-term potentiation (green) and long-term depression (blue) are reported as the baseline I_D (V_D=-0.2 V_G=0) after the application of the given gate voltage to an initiated channel for 30 seconds. The long-term depression (blue) is reported as the baseline I_D at the same conditions after the application of the given gate voltage for 1 second.

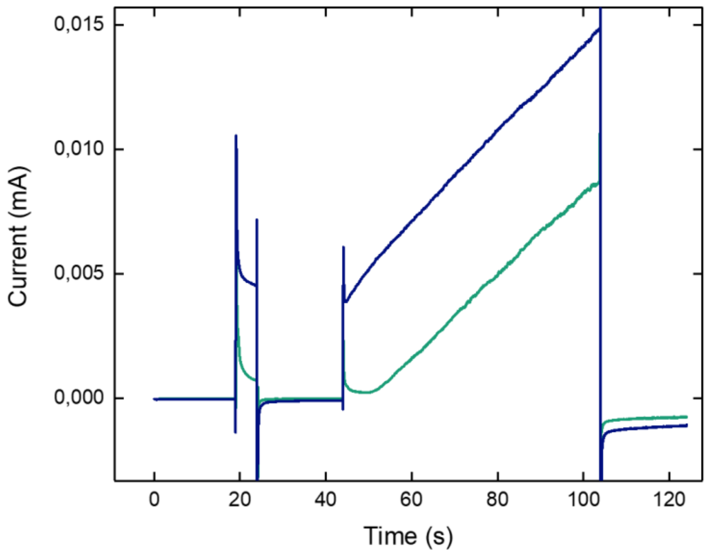

Figure S3. Repeated ETE-S electrodeposition. We demonstrate the reusability of a set of gold source and drain electrodes after over-oxidation of the PETE-S channel. The following V_D sequence was used to grow the channel in two steps and evaluate the conductance after each step: -0.2 V/20 s; 1 V/5s; -0.2 V/20 s; 0.5 V/60 s; -0.2 V/20 s. The resulting plot of I_D vs. time at a V_G of 0V in the presence of 1 mg/mL ETE-S represents PETE-S deposition across a pristine set of electrodes (green) and across the same set of electrodes after the existing polymer is over-oxidized by applying a V_G of -1.5 V for 2.5 s (blue).

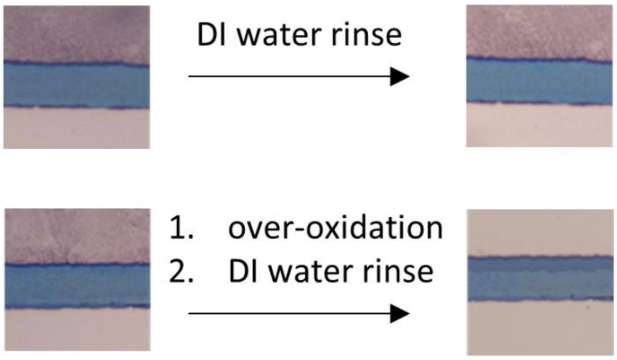

Figure S4. Localized over-oxidation. Optical micrographs of two identically made transistor channels (left), which are subjected either to a DI water rinse or to over-oxidation, followed by a DI water rinse. After rinsing, a gap clearly forms between the electrode at the top of the image and the active channel material.

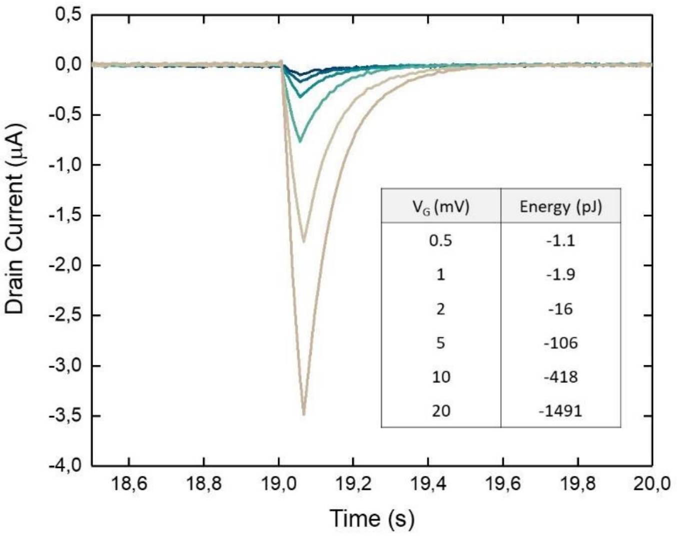

Figure S5. Minimizing energy consumption. The characterization pulse was minimized with respect to gate voltage and pulse duration to reduce the amount of energy required. Upon the application of -0.5 mV for 50 ms, the energy required to produce a characterization pulse can be reduced to 1 pJ.

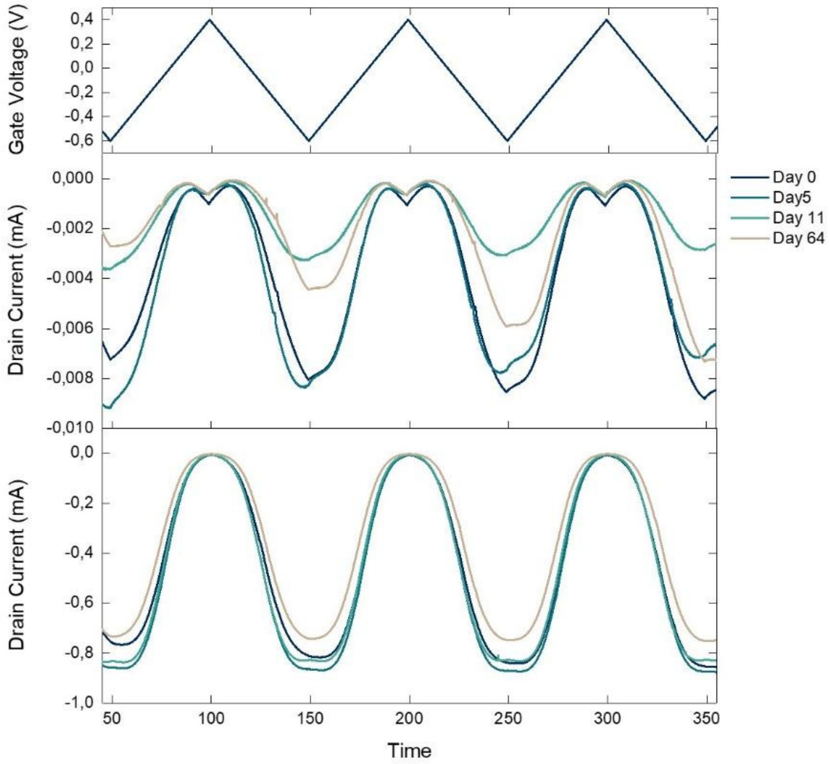

Figure S6. Device stability. Transfer curves at a drain voltage of -0.2V and a varying gate voltage (top graph) were obtained at four timepoints for two devices. One device was fabricated by applying a drain voltage of -1 V for 5 seconds, followed by a drain voltage of -0.5 V for 30 seconds (bottom graph). The second device was fabricated identically and then over-oxidized by applying a gate voltage of -2 V for 10 seconds. Devices were stored dry in the dark at room temperature in the lab environment.

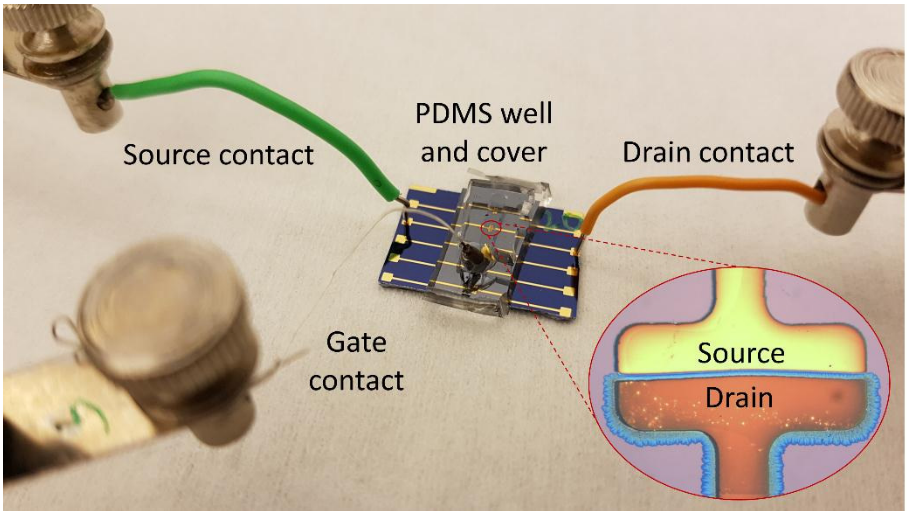

Figure S7. Experimental setup. Gold source and drain electrodes separated by a 30 μm gap were thermally evaporated on a silicon substrate using a shadow mask. An SP-300 Bio-Logic potentiostat/galvanostat in a synchronized two-channel configuration was used for time-resolved measurements (Bio-Logic Science Instruments, France). The source contact was connected to the ground, counter, and reference leads of both channels while the working leads were connected to the drain and gate electrodes. An Ag/AgCl pellet electrode was used as the gate (Warner Instruments, USA). The inset shows an optical microscopy image, taken using a 10x objective, of a PETE-S channel spanning a 30 μm gap between the gold source and drain electrodes. The PETE-S was grown by applying a potential of 1V to the drain for 30 seconds.

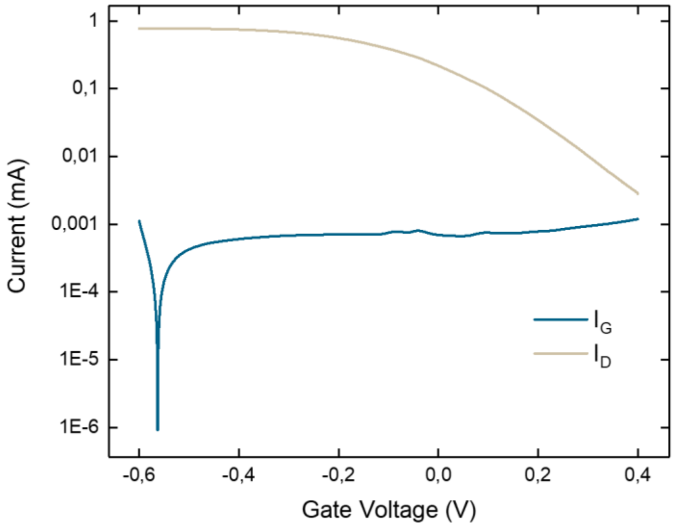

Figure S8. Leakage current. The leakage current at the gate (I_G) is compared to the drain current (I_D) within the operating gate voltage range.

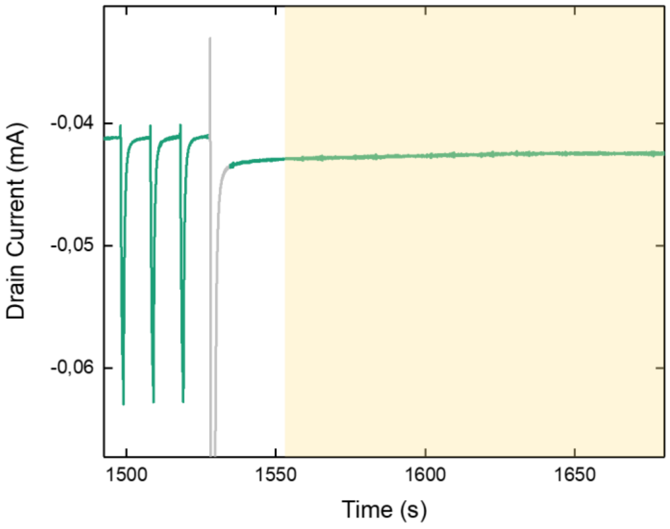

Figure S9. LTP retention. The highlighted section of data, obtained for 2 minutes at a V_G of 0V at the tail end of the LTP sequence presented in Figure 4a, exhibits a decay of less than 0.3% per minute.

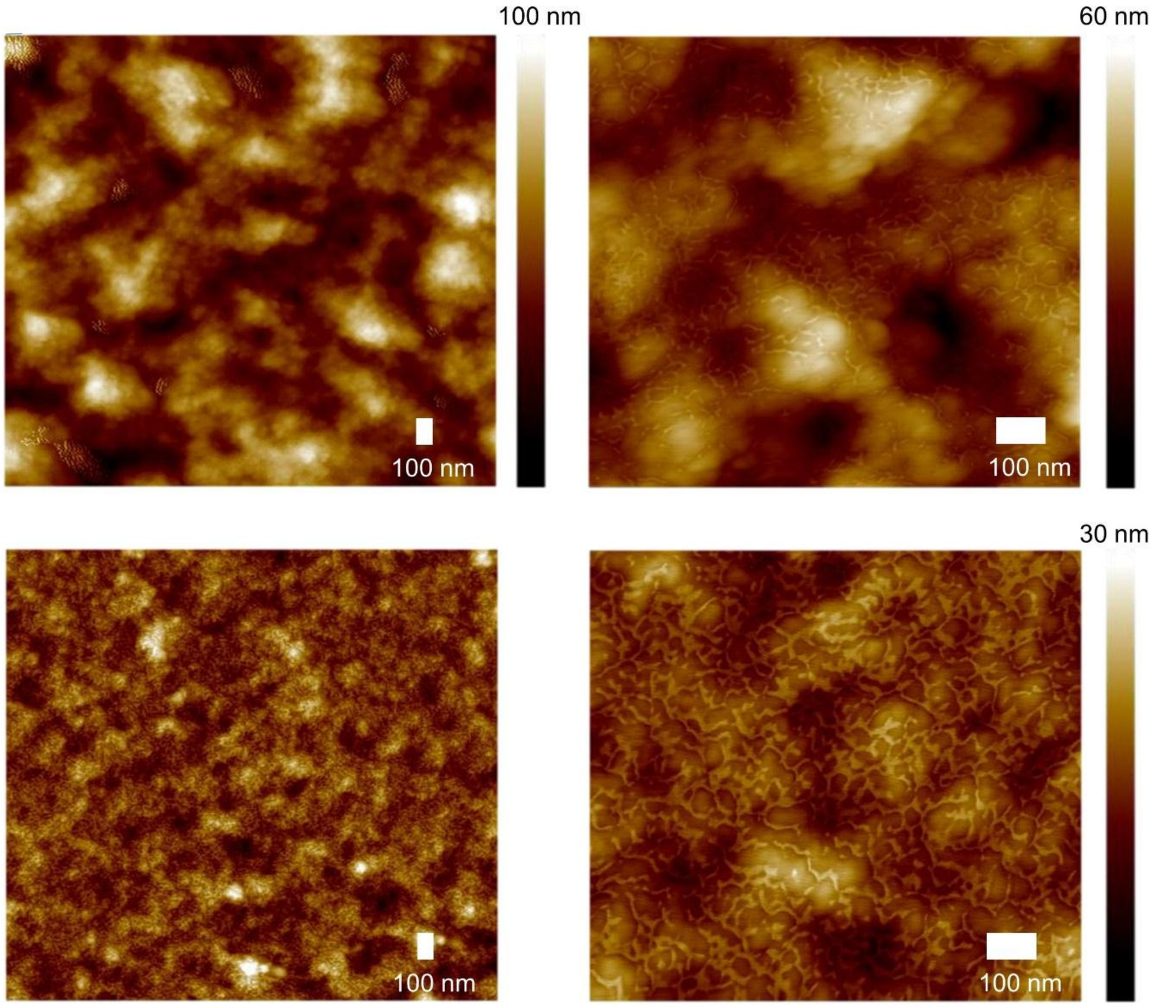

Figure S10. Atomic force microscopy. AFM images of the drain (top) and source (bottom) electrodes, obtained in tapping mode. The OECT depicted here is the same as is shown in Figure 4**.**

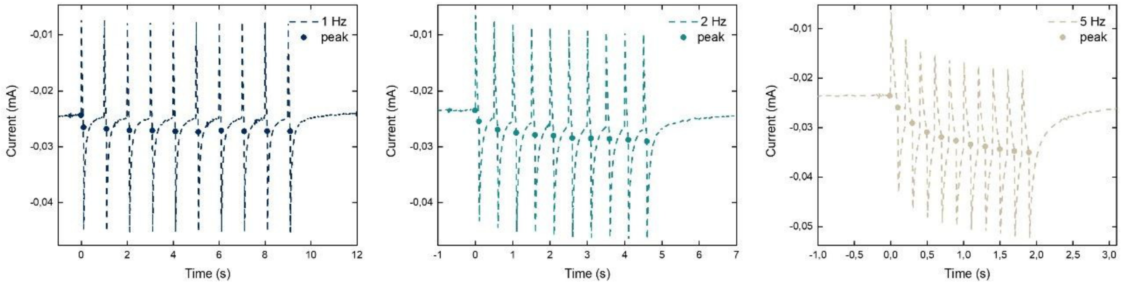

Figure S11. Short-term potentiation in the time domain. The gate voltage spikes with an amplitude of -0.1 V and a duration of 100 ms are applied at different frequencies. The amplitude of the drain current spike (shown as points) is taken as the drain current intensity at the last measured point before the gate voltage switches back to 0 V.

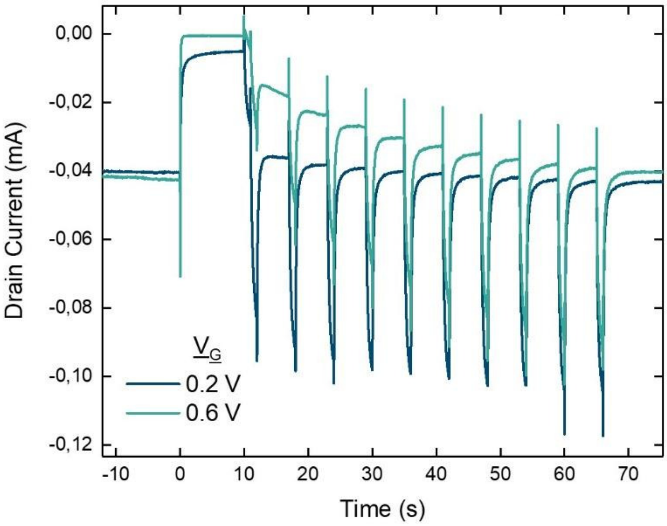

Figure S12. Short-term depression in the time domain. The gate voltage spikes with an amplitude of -0.1 V and a duration of 1 s are applied following a positive gate voltage spike of varying magnitudes. The amplitude of the drain current spike is taken as the drain current intensity at the last measured point before the gate voltage switches back to 0 V.

V_{In1}	V_{In2}	Device
on	off	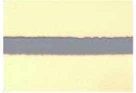
off	on	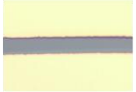
on	on	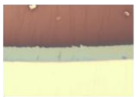

Figure S13: Optical micrograph to provide validation of the device described in Figure 6. Neither the input from channel 1 (V_{In1}) nor the input from channel 2 (V_{In2}) is sufficient to induce electropolymerization on its own. When both inputs are applied, however, polymerization is initiated.
